# A Prolapsing Vitello-intestinal Duct in Newborn

**Published:** 2013-07-31

**Authors:** Nitinkumar Borkar

**Affiliations:** Department of Trauma and Emergency, AIIMS Raipur Chhattisgarh, India, 492099

Pediatrician attending to a newborn delivered by emergency cesarean section observed a red coloured mass protruding from the umbilicus by the side of umbilical cord. Pediatric surgeon was called for opinion. Pediatrician had tied the umbilical cord with a sterile thread rather than usual umbilical clamp. It was a full term male baby with a patent vitello-intestinal duct (VID) and a prolapsing proximal and distal intestinal loop giving shape of an inverted Y (Fig. 1). Meconium was seen coming from one of the limbs of the Y. Antenatal checkup and all antenatal ultrasound scans were reported as normal. Patient was kept under observation and planned for early intervention. Under general anesthesia, prolapsed loop was approached through circum-umbilical incision at mucocutaneous junction after reducing the prolapsed part. Intestine was delivered outside the abdomen; vitellointestinal duct opening was in distal ileum as opening was too wide so that resection and anastomosis was done. Intestine replaced inside the abdomen and umbilicoplasty was done with absorbable sutures. Postoperative period was uneventful and patient discharged on 5th postoperative day.

**Figure:**
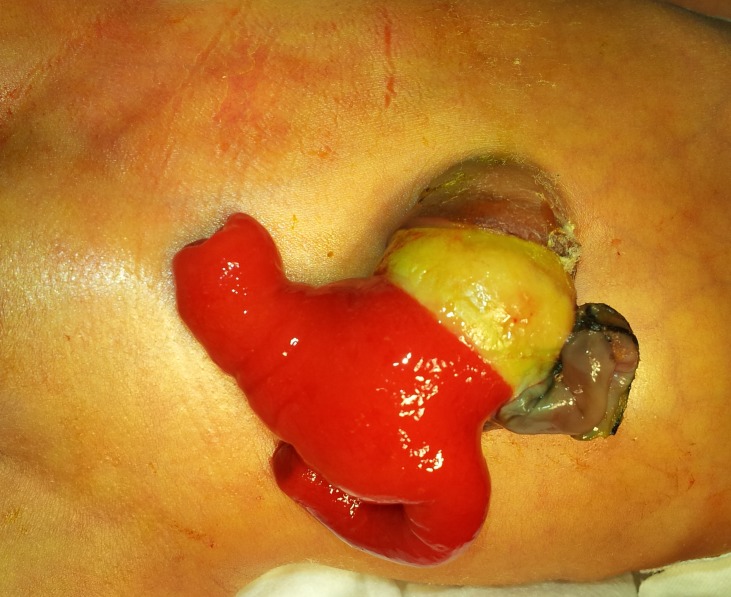
Figure 1:Prolapsed distal and proximal loop of patent VID with umbilical cord by its side

## DISCUSSION

Remnants of vitello-intestinal duct account for a wide variety of umbilical abnormalities. These include fistula, sinus tract, umbilical adenoma, enterocystoma and congenital bands.[1] Incidence of patent VID is reported as 0.0053%.[2] Patent VID is an infrequent but well known anomaly.[3] Bowel prolapse with patent VID is rare and prolapsing proximal as well as distal loop in a newborn is extremely rare. Although Meckel’s diverticulum is the most common VID anomaly, patent VID is the most common symptomatic embryological defect. [4, 5] Patient may present with the anomaly itself or due to complications like intestinal obstruction secondary to volvulus, intussusceptions or adhesions. Complications of patent VID may be minor like feculent discharge leading to periumbilical skin excoriation. Prolapse occurs if defect is wide enough to allow bowel to come out or due to increased intra-abdominal pressure like cry or cough. Prolapse can be partial or complete. The condition requires identification and early management to avoid complications. Cord tying in such neonates should be done with great care using sterile thread to prevent injury to the prolapsed bowel. Resection anastomosis following reduction of the prolapse loop by a circum-umbilical incision is the treatment of choice though other surgical approaches may be adopted.

## Footnotes

**Source of Support:** Nil

**Conflict of Interest:** None declared

